# Transposable Elements and Human Diseases: Mechanisms and Implication in the Response to Environmental Pollutants

**DOI:** 10.3390/ijms23052551

**Published:** 2022-02-25

**Authors:** Benoît Chénais

**Affiliations:** BiOSSE (Biology of Organisms: Stress, Health, Environment), UFR Sciences et Techniques, Le Mans University, F-72085 Le Mans, France; bchenais@univ-lemans.fr

**Keywords:** transposable element, cancer, metabolic disease, neurologic disease, aging, common disease, environmental pollutants, epigenetics, DNA methylation, DNA repair

## Abstract

Transposable elements (TEs) are recognized as major players in genome plasticity and evolution. The high abundance of TEs in the human genome, especially the *Alu* and Long Interspersed Nuclear Element-1 (LINE-1) repeats, makes them responsible for the molecular origin of several diseases. This involves several molecular mechanisms that are presented in this review: insertional mutation, DNA recombination and chromosomal rearrangements, modification of gene expression, as well as alteration of epigenetic regulations. This literature review also presents some of the more recent and/or more classical examples of human diseases in which TEs are involved. Whether through insertion of LINE-1 or *Alu* elements that cause chromosomal rearrangements, or through epigenetic modifications, TEs are widely implicated in the origin of human cancers. Many other human diseases can have a molecular origin in TE-mediated chromosomal recombination or alteration of gene structure and/or expression. These diseases are very diverse and include hemoglobinopathies, metabolic and neurological diseases, and common diseases. Moreover, TEs can also have an impact on aging. Finally, the exposure of individuals to stresses and environmental contaminants seems to have a non-negligible impact on the epigenetic derepression and mobility of TEs, which can lead to the development of diseases. Thus, improving our knowledge of TEs may lead to new potential diagnostic markers of diseases.

## 1. Transposable Elements of the Human Genome

Like many other eukaryotic genomes, the human genome is littered with ancestral traces of transposable element (TE) invasion. These TEs and TE fragments now represent nearly half of the genome [1,2]. Most of these TE insertions have lost their ability to propagate in the genome, but all of these TEs remain a source of chromosome recombination and mutations. As described elsewhere, TEs are diverse in nature and two main classes are identified based on their mechanism of transposition [3]. Class I elements, retroelements or retrotransposons, propagate by reverse transcription of an RNA intermediate. Class II elements, or DNA transposons, move primarily by a “cut-and-paste” mechanism involving excision and reinsertion of their DNA sequence. In addition, a large number of non-autonomous repeat sequences are affiliated with TEs but are dependent on the enzyme(s) encoded by an auxiliary element, which is itself an autonomous TE, to ensure their mobility and amplification in the genome. Short Interspersed Nuclear Elements (SINEs) elements are the major class of these non-autonomous TEs that have widely invaded the human genome [2].

**Figure 1:**
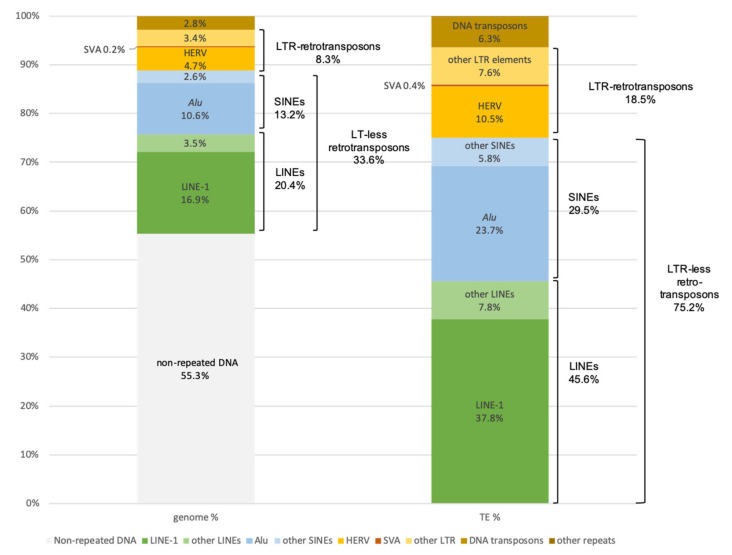
Proportions of transposable elements (TEs) in the human genome and their distribution in different families and subfamilies. Left panel: the percentage of each class or subclass of TEs is indicated with respect to the whole genome according to data from [1]. Right panel: the distribution of TEs in each category is given as a percentage of the total TEs present in the genome according to [1].

**Figure 2:**
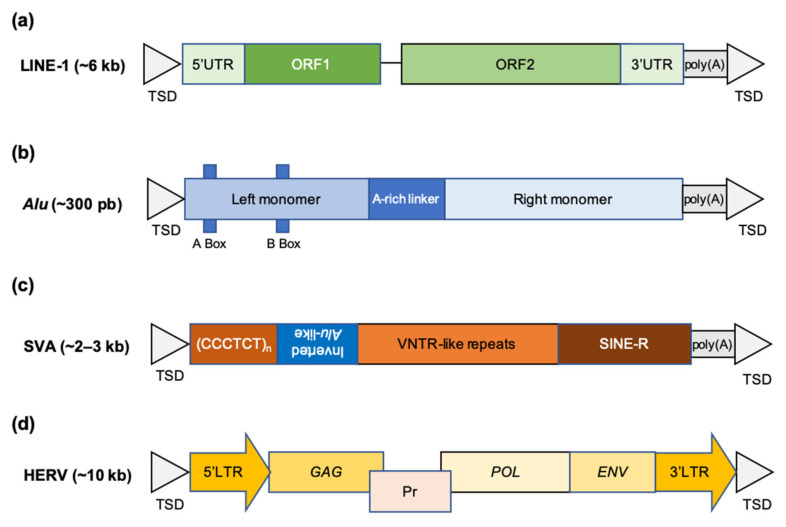
Schematic structure of the main human TEs of class I (retroelements). (**a**) The long terminal repeats (LTR)-less retrotransposon LINE-1 is commonly constituted of two open reading frames (ORF-1 and -2) with untranslated regions (UTR) on both sides and a 3′ end poly(A) tail. The sequence is flanked by target duplication sites (TSD). (**b**) The non-autonomous LTR-less retroelement *Alu* usually harbors two monomers separated by a A-rich linker. *Alu* also displays a poly(A) tail and TSDs. (**c**) SVA elements are non-autonomous composite TEs classically constituted of a C-rich repeat, inverted *Alu*-like sequence, VNTR-like repeats and SINE sequence. (**d**) The classical structure of the main human LTR-retrotransposons HERVs includes TSDs, 5′ and 3′LTR, the three retroviral ORFs (*GAG*, *POL*, *ENV*) and an additional ORF (Pr).

Class I elements are by far the most numerous in the human genome. They are subdivided into retrotransposons with long terminal repeats (LTRs) and retrotransposons without LTRs. The latter are very predominant, and 75.2% of human TEs are LTR-less retrotransposons, representing alone at least 33.6% of the whole human genome [1] (Figure 1). Among these LTR-less retrotransposons, the Long INterspersed Nuclear Element-1 (LINE-1 or L1; Figure 2a) has undergone an exceptional expansion and now counts about 500,000 copies, mostly fragmented. This accounts for 38% of human TEs and 16.9% of the entire genome (Figure 1). However, only a very small fraction of these LINE-1s is active, i.e., capable of transposition, which means being able to propagate through the reverse transcription of an RNA intermediate and insertion of this DNA at another location in the genome. Thus, about 100 sequences among the L1PA1, L1PA2 subfamilies or *Homo sapiens*-specific LINE-1s (L1Hs) are active [4].

The second most frequent category of TEs is the non-autonomous SINEs, in particular the *Alu* repeats (Figure 2b), which correspond to 24% of TEs and 10.6% of the genome (Figure 1). These *Alu* repeats are present in nearly one million copies in the entire human reference genome [1] and are derived from a few young *Alu* subfamilies (*Alu*Y), traces of an explosion of *Alu* retrotransposition in ancestral primates dating back 35 to 40 million years [5]. The *Alu* sequences are dependent on the endonuclease and reverse transcriptase activities of the LINE-1 element for propagation in genomes. Other SINEs found in humans are the Mammalian-wide Interspersed Repeat (MIR) elements and, in particular, MIR3. Other non-autonomous elements composed of a composite assembly of sequences homologous to SINEs, Variable Number of Tandem Repeats (VNTR) and *Alu* sequences, hence their name SVA (for SINE, VNTR, *Alu*; Figure 2c), are mobilizable, i.e., can be moved by the enzymes of LINE-1 elements. There are a limited number of subfamilies of *Alu* and SVA elements capable of mobilization in present-day humans: for example, *Alu*Ya5, *Alu*Yb8, *Alu*Yb9, SVA-E and SVA-F. The LINE-1 elements are active in the germline making LINE-1, *Alu* and SVA integrations common and segregating DNA markers in populations [4,6,7].

The LTR retrotransposons are present in smaller numbers in the human genome and are mainly represented by Human Endogenous RetroVirus (HERVs; Figure 2d), which resemble retroviruses in both structure and mechanism of mobility but lack functional envelope genes. In humans, the activity of LTR retrotransposons has been nearly extinguished over the last few million years [8], although their sequences still represent about 8% of our genome (Figure 1). The various HERVs are named according to the transfer RNA that initiates their reverse transcription reaction; the most recent active subfamily is HERV-K (initiated by the lysine transfer RNA). There are also HERV-I (initiated by isoleucine tRNA) and HERV-L (initiated by leucine tRNA) sequences. The current human genome still has insertion polymorphisms for these HERV elements. LTRs flanking full proviral insertions tend to recombine, removing intervening sequences and reducing the insertion to a solo LTR [9].

Class II elements, or transposons, are much less numerous in the human genome, accounting for only 6% of TEs and 2.8% of the entire genome (Figure 1). They mainly belong to the following three superfamilies: (i) TC-1/mariner, i.e., mariner, MER2-Tigger, and Tc2; (ii) hAT, i.e., MER-1-Charlie and Zaphod; and (iii) PiggyBac [1].

TEs are an evolutionary force that contributes to the genetic diversity of organisms, especially when they are recruited to the host genome to become new genes; this domestication of TEs is a source of genetic innovation [10,11]. However, transposition activity can also contribute to the emergence of human diseases and the insertion of a TE into the genome has several direct or indirect consequences on the structure of the genome and the control of gene expression.

## 2. TE Insertion and Its Consequences on the Genome and Gene Expression

Regarding events that can lead to disease in humans, three major groups of mechanisms can be distinguished: (1) genomic rearrangements due to TE abundance; (2) modification of gene structure and regulatory regions by TE insertion, and (3) alteration of epigenetic controls [4,12,13].

### 2.1. Chromosome Rearrangements

**Figure 3:**
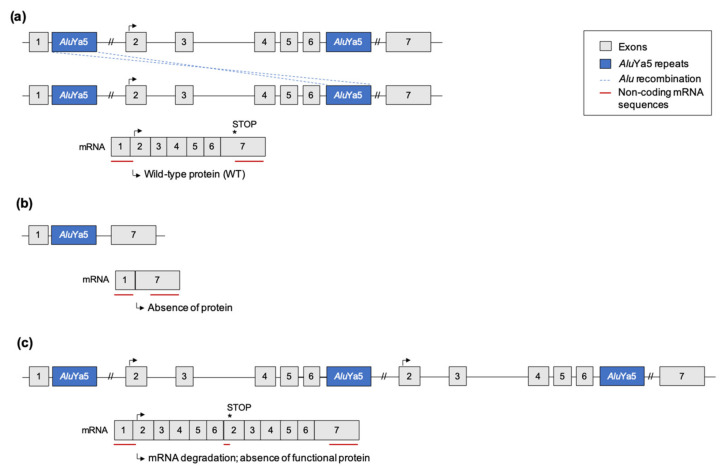
Insertion of *Alu* elements and recombination. Example of recombination between two *Alu*Ya5 insertions in the parental alleles of the *UBE2T* gene (**a**), leading to one recombination allele with deletion (**b**) and one recombination allele with duplication (**c**). The exons are represented by grey boxes and the *Alu*Ya5 insertions by blue boxes. Figure adapted from [14].

The high abundance of LINE-1 and *Alu* repeats (approximately 17% and 11% of the human genome, respectively) favors ectopic recombination, i.e., recombination between non-homologous loci. Such recombination often results in significant chromosomal rearrangements such as gene deletions, duplications, translocations or chromosomal inversions that are the source of pathologies (as described in the above paragraphs 3 and 4). An example of this type of recombination between two non-allelic insertions is given in Figure 3. In this case, *Alu-Alu* recombination occurred between tandem *Alu*Ya5 insertions at the *ubiquitin E2 T conjugating enzyme* (*UBE2T*) gene causing Fanconi anemia. The rearrangement causes an interstitial deletion encompassing exons 2–6 of the paternal allele, with a corresponding duplication of the maternal allele in the patient [14].

Indeed, LINE-1 and *Alu* elements are frequently observed in or near breakpoints of chromosomal rearrangements, and LINE-1 alone accounts for 19% of the 2081 breakpoints analyzed in a dataset of 17 entire human genomes [15]. Although present in smaller numbers in the human genome, DNA transposons can also be a source of chromosomal rearrangement. For example, the MER20 transposon is found on chromosome 19 and appears to be most notably linked to the development of acute lymphoblastic leukemia among the 10 different leukemias or lymphomas studied [16].

### 2.2. Modification of the Structure and Expression of Genes by the Insertion of ETs

Another way for TEs to have an impact on the human genome and to induce diseases is to modify the structure of genes and their regulatory regions. Indeed, de novo insertion, i.e., acquired directly in the patient or a mosaic parent, or an insertion inherited from the ascendants as a classical allele (i.e., insertion allele) can be responsible for diseases that behave like classical monogenic diseases. For this to happen, the TE insertion must appear in the germline and critically affect the function of the gene. The TE insertion will therefore create a new allele, which can be either a loss of function allele or a gain of function allele.

The insertion of a TE into a gene can have several consequences, including the creation of new exons or new introns, which are called exonization and intronization, respectively. The creation of a new exon causes a reading frame shift and/or creates premature stop codons in about 79% of cases [17]. As a result, the resulting mRNA are either prematurely degraded or aberrantly spliced, and if it manages to be translated into a protein, it is truncated and non-functional. Thus, exonization events following the insertion of LINE-1 or *Alu* elements can cause human diseases as described below.

**Figure 4:**
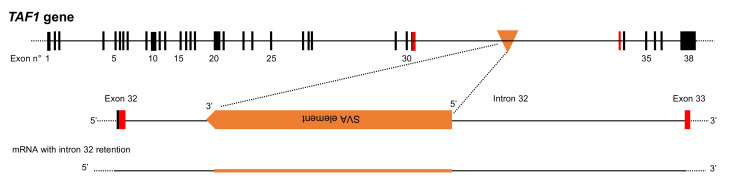
Example of SVA element insertion causing X-linked dystonia with parkinsonism in the *TAF1* gene. *TAF1* consists of 38 exons (vertical bars; red bars are exons flanking the insertion) with a SVA insertion in reverse orientation in intron 32 (orange triangle). This insertion leads to the retention of intron 32 in the mRNA and the variable number of repeats of the hexanucleotide (CCCTCT)n (see Figure 2c) affects the disease.

TE insertion can also alter mRNA splicing and cause aberrant alternative splicing leading to exon skipping or intron retention. For example, the insertion of an SVA element into an intron of the *TATA box-binding protein-associated factor 1* (*TAF1*) gene causes X-linked dystonia with parkinsonism (XDP), a movement disorder endemic to the Philippines. The presence of the SVA element prevents normal splicing of *TAF1* mRNA and results in retention of intron 32 [18] (Figure 4). However, the real situation is more complex, and a range of alleles exist in the population with varying size expansions of the hexanucleotide repeat domain (CCCTCT)n within the SVA. The length of this repeat is strongly correlated with disease onset, and longer repeat sequences are associated with earlier onset of symptoms [19]. Although the precise role of the length of this hexanucleotide repeat is not entirely clear, it affects the efficiency of retroelement transcription and promotes guanine stacking, a DNA structure known as a G-quadruplex [19].

**Figure 5:**
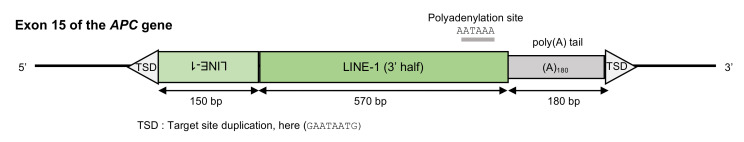
Insertion of a LINE-1 element into the *APC* gene. The insertion of a truncated and partially inverted LINE-1 element in the last exon of the *APC* gene results in a new polyadenylation site.

As LINE-1 elements include a polyadenylation signal in their own sequence, a polyadenylation signal (i.e., the sequence AATAAA) is frequently created in the A-rich tails of SINE and LINE elements. Therefore, human retroelements, such as LINE-1, *Alu*, or even HERVs that contain a polyadenylation signal in their LTRs, can introduce intragenic polyadenylation signals, creating new mRNA isoforms. These alternative mRNAs are often truncated by the loss of one or more exons. This modification of polyadenylation sites has been observed in several human diseases as described below. Figure 5 shows the example of the insertion of a LINE-1 element (truncated and partially inverted) in the last exon of the *APC* gene, causing colon cancer and desmoid tumors (or aggressive fibromatoses) [20].

**Figure 6:**
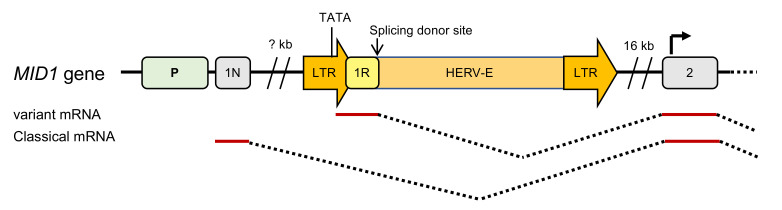
Example of the human endogenous retrovirus (HERV)-E element insertion causing X-linked Opitz syndrome. Schematic structure of the 5′ part of the *MID1* gene, including the promoter (P, green box) and the first two introns (1N and 2, grey boxes). The insertion of the HERV-E element brings a new transcription start site (TATA) and a new intron 1 (1R, yellow box) leading to an alternative transcript (mRNA).

Finally, the use of regulatory elements provided by the TE insertion is a way to modify gene expression. In particular, the promoter or enhancer/silencer elements of HERV LTRs are particularly powerful. For example, the X-linked form of Opitz syndrome is associated with the presence of an HERV-E element whose LTR acts as a tissue-specific promoter and activator of the *MID1* gene [21] (Figure 6).

### 2.3. Escape from Epigenetic Control

Because TEs can be considered a threat to genome integrity, host species have developed defense mechanisms to silence them. These mechanisms rely on epigenetic regulatory pathways such as DNA methylation and inhibitory modifications of histone proteins that are achieved, in the case of retrotransposons, through PIWI-interacting RNA (piRNAs) and various protein complexes, as detailed elsewhere [22,23]. In addition, the evolutionary conserved molecular chaperone heat-shock protein-90 (HSP90) also prevent TE jumping and mutagenic effect through its interaction with Piwi proteins [24].

However, these silencing mechanisms are imperfect and some TEs manage to bypass this regulation and are transcribed. For the oldest and/or non-coding TEs, the consequences of this situation may be unimportant but for active TEs, i.e., able to move, a transcription of the TE may lead to new insertions. Nevertheless, even transcripts of a priori inactive elements can have an impact on genome expression in the form of long non-coding RNAs (LncRNAs). Moreover, the relationship between TEs and epigenetic control is two-way, and if DNA hypomethylation is a cause of TE activation, the presence of TEs in a genomic region is also a cause of chromatin hypermethylation. Thus, epigenetic modification of TE regulatory sequences can impact the expression of neighboring genes [25,26,27].

A correlation between TE activity and chromatin regulation has been observed, primarily at the level of DNA methylation, and DNA hypomethylation is considered a facilitator of TE mobility. As chromatin methylation occurs preferentially at CG dinucleotides called CpG (Cytosine-phosphate-Guanine) islands, the relationship between pathological markers and the methylation status of whole genome CpG islands on the one hand, and LINE-1 elements on the other hand, has been particularly studied in cancers [28,29,30]. Thus, by analyzing the whole genome of cancer cells of diverse tissue origins, it is possible to observe a preference for insertion of LINE-1 elements in DNA regions with hypomethylation and hypomethylation of the TEs themselves and thus their derepression [31,32].

### 2.4. Interaction of TEs with DNA Repair Pathways

Transposition events generate different types of DNA damage, including single-base mismatches and double strand breaks (DSB), which lead to the activation of DNA damage repair pathways [33]. The transcriptomic study of Wang and Liang in osteosarcoma patients highlighted the correlation between the upregulation of TE expression and the overexpression of 29 genes involved in homologous recombination (HR), non-homologous end joining (NHEJ), and DNA mismatch repair pathways [34]. However, DNA repair actors can also act as inhibitors of LINE-1 transposition, suggesting a complex interplay between transposition and DNA damage repair pathways.

Indeed, the regulation of LINE-1 expression and activity is rather complex and includes, but is not limited to, epigenetic controls and interaction with several DNA repair proteins that act as inhibitors of LINE-1 integration in the genome (reviewed in [35]). For example, core proteins of the nucleotide excision repair (NER) pathway, XPD and XPA, the lesion binding protein, XPC, and the endonuclease complex ERCC1-XPF, limits LINE-1 retrotransposition. However, NER proteins may also play a role in the normal LINE-1 insertion process as suggested by the presence of abnormally large duplications at the insertion site in NER-deficient cells [36]. The retrotransposition of LINE-1 is also controlled by proteins involved in postreplicative DNA repair and HR such as ATM and BRCA1. In the case of ATM, the lack or a strong decrease in ATM kinase leads to an increase of LINE-1 activity in neural stem cells [37]. On its side, BRCA1 causes the formation of a target site duplication by initiating double-stranded cleavage, resection and protective coating of DNA ends by replication proteins A, which prevents LINE-1 insertion into the replication fork [38]. The BRCA1 depletion in cells increases LINE-1 activity [38]. In addition, cytoplasmic BRCA1 can inhibit the translation of the open reading frame (ORF)-2 of LINE-1 and the formation of functional LINE-1 ribonucleoprotein [38]. An increased LINE-1 expression and activity was reported in case of defect in Fanconi anemia factors, SLX4/FANCP and FANCD2 [39] In addition, FANCD2 activates the ubiquitin ligase RAD18 leading to the monoubiquitination of the proliferating cell nuclear antigen protein, which interacts with LINE-1 ORF2 during retrotransposition and may therefore inhibit LINE-1 insertion [40]. An interaction of RAD18 with ORF1 has also been found and leads to the formation of P-bodies and stress granules [40]. Finally, overexpression of RAD18 strongly suppressed LINE-1 activity as well as LINE-1-mediated *Alu* retrotransposition [40].

The high abundance of *Alu* elements in the human genome creates a major source of non-allelic homologies, which influence DSB repair and lead to altered forms of genetic instability through *Alu*-related repeat mediated deletion (RMD) [41,42]. In addition to their repetitive nature, mismatches between *Alu* elements also increase the complexity of their interaction with DNA repair processes. Thus, *Alu*/*Alu*-related RMD, which relies on the HR mechanism, is the primary repair mechanism in the absence of divergence between *Alu* sequences, while an alternative NHEJ process takes over when mismatches increase [41,42]. This so-called homeologically induced RMD involves the DNA mismatch repair pathway [42], whereas homology-dependent RMDs, which occur with increased sequence homology, are mainly RAD52-dependent [41]. These data strongly suggest that sequence divergence between the involved *Alu* elements is a critical selection factor between several competing DNA repair processes [41,42].

## 3. Involvement of TEs in Human Cancers

Whether through the insertion of LINE-1 or *Alu* elements that cause chromosomal rearrangements or through epigenetic modifications, TEs are widely implicated in the origin of human cancers [29,30,43,44,45,46,47,48]. The role of TEs as a source of genomic instability to induce cancer was also demonstrated experimentally in the zebrafish model of melanoma [49]. The results highlighted a specific upregulation of a LINE and an LTR element in malignant melanomas of transgenic fish. Although this has to be confirmed, TEs expression and methylation levels could be used as prognostic markers [50,51]. Indeed, the quantification of *Alu* sequence and of LINE-1 methylation may distinguish lung or breast cancer patients from healthy patients [50]. Whole genome sequencing (WGS) of several cancer tissues, i.e., 43 cancer samples from either colorectal, prostate, ovarian, multiple myeloma, and glioblastoma origin, and their matched normal blood samples, pinpointed that LINE-1 and *Alu* somatic insertions were more frequently observed in cancers of epithelial origin (e.g., colorectal, prostate, and ovarian) compared with blood or brain cancers [52]. Similarly, a previous study of 244 patients with 12 types of cancers showed that colorectal and lung cancers were the most frequently affected by LINE-1 somatic insertions (93% and 75% of patients, respectively) and that, overall, 53% of patients had at least one somatic insertion of LINE-1 [32]. Furthermore, insertion of TEs (i.e., LINE-1, *Alu*, SVA, and HERV) was studied in 202 colorectal tumors and the average number of somatic insertions was estimated to be 25, but with high variability among individuals [53]. Recently, Wang and Liang conducted an integrative analysis of WGS, RNA-seq and methylation data in osteosarcoma patients (39, 37 and 36 patient samples, respectively) showing upregulation of LINE-1, *Alu*, SVA and HERV-K [34]. About 80% of these TE insertions in osteosarcoma are germline insertions. However, among 68 cancer-associated genes (i.e., 32 oncogenes and 36 tumor suppressor genes), 15 have osteosarcoma-specific insertion (Table 1), mainly occurring in transcription factor genes involved in cancer development and neuronal process genes [34]. This study also showcases that three of the 34 patients analyzed for event-free survival had more than 100 TE insertions, suggesting that the greater the number of TE insertions, the longer the event-free survival time [34]. Finally, in colorectal cancer patients, the expression level of some TEs, i.e., nine among SVA, SINE, LINE and HERV families, was correlated with decreased survival [51]. In addition, tumors with the highest TE expression level showed increased immune cell infiltration with upregulation of interferon signaling pathways and increased expression of PD-L1 in immune cells [51].

### 3.1. LINE-1 Insertion

About 50% of human tumors contain somatic retrotranspositions of LINE-1 elements [32]. The first example of a LINE-1 element insertion was reported as early as 1988 by Morse et al., showing the insertion of a LINE-1 retrotransposon into the human proto-oncogene *c-MYC* of human breast carcinoma cells (Table 1) [54]. Comparison of the *c-MYC* gene structure of cancer tissue cells and non-cancerous breast cells from the same patient revealed, in the tumor cells, a specific rearrangement of one of the *MYC* loci and amplification of the second *MYC* locus located on the homologous chromosome. A LINE-1-like sequence was found in the second intron of the rearranged *MYC* locus. In this case, the LINE-1 sequence functioned as a mobile genetic element to induce a somatic mutation [54]. Another example of somatic insertion of the LINE-1 element was observed in the *APC* tumor suppressor gene (Figure 4) in several patients with colon cancer and in association with desmoid tumors or aggressive fibromatoses [20]. Whole genome sequencing and transcriptomic data are very useful to study insertions and transcription of TEs, respectively. In this way, a high level of LINE-1 transcription as well as an increase in LINE-1 insertion activity was found in three cancer types, namely primary tumor samples of invasive breast carcinoma, head and neck squamous cell carcinoma, and lung adenocarcinoma [55]. More recently, a genomics study of a set of nearly 3000 tumor samples covering 38 different cancer types highlighted the importance of LINE-1 elements and the genomic remodeling they induce [56]. Indeed, out of nearly 20,000 retrotransposition events observed in these cancer samples, 98% are due to a LINE-1 element while *Alu* and SVA elements are only in the very small minority [56]. Finally, by examining four metastatic breast cancer patients, Steely et al. have identified 11 TE insertions or associated structural variants, including three LINE-1, six *Alu*, and two SVA, and found that the majority of these occurred early in tumor progression [57]. Three main events have been identified: a LINE-1-mediated intergenic deletion, a LINE-1 intronic insertion, and a LINE-1 insertion in an unspecified exon of *NIPAL2* gene [57].

### 3.2. Insertion of Alu Sequences and Chromosomal Recombination

As previously mentioned, *Alu* repeats represent 11% of the human genome. These repeats are at the origin of non-allelic homologous recombination events, or ectopic recombination, leading to several cancerous diseases (Table 1). These recombination events have several consequences and can lead to chromosomal translocations, deletions or duplications [12,46]. The insertion of an *Alu* sequence or a deletion resulting from the recombination between *Alu* sequences can themselves generate an exon skipping [12,46].

Atypical teratoid/rhabdoid tumor (AT/RT) is a malignant brain tumor that occurs most commonly in infants and is often associated with a germline alteration of the *SMARCB1* gene. A case was recently reported with an *AluY* insertion in exon 2 of the *SMARCB1* gene that caused a frameshift truncation absent in the patient’s germline [58].

The *BRCA1* and *BRCA2* genes, which are associated with breast and ovarian cancers, are important sites of *Alu* element insertion and an unusually high density of *Alu* repeats is found at these loci. Numerous deletion events have also been reported at the *BRCA1* locus and, to a lesser extent, for the *BRCA2* gene (reviewed in [46,59]). In addition, one of the first germline mutations caused by a TE to be identified is the insertion of an *Alu* element in exon 22 of the *BRCA2* gene. This insertion results in alternative splicing and skipping of exon 22 in the transcribed RNA [60]. As presented above, Steely et al. searched for a TE insertion in four patients with metastatic breast cancer and found five *Alu* insertions or associated deletions, all of which are intergenic. Only one event was intragenic, namely an *Alu*-mediated translocation within the third intron of the *MAP2K4* gene (17:11,974,341/22:48,343,831) [57].

Many other cases of deletions caused by recombination between *Alu* elements have been described [4,12,13,46]. For example, homologous recombination between *Alu* sequences at the *Caspase-activated DNase* (*CAD*) gene is frequently detected in human hepatoma tissue. It leads to a deletion of exon-3 which, in turn, leads to the skipping of this exon or its replacement by a partial *Alu* sequence, producing in both cases a CAD protein truncated in its C-terminal part [61]. A statistically significant overrepresentation of *Alu* elements around the breakpoints can also be observed in deletions of the *cadherin-1* (*CDH1*) gene [62]. These large deletions are found in 4% of families with hereditary diffuse gastric cancers and thus seem to originate from homologous recombination between *Alu* repeat sequences [62]. It should also be noted that the insertion of *Alu* elements in the *von Hippel-Lindau* (*VHL*) gene is involved in the deletions that cause von Hippel-Lindau disease, an autosomal dominant syndrome that predisposes to the development of benign or malignant tumors [63].

Homologous recombination between *Alu* elements sometimes results in gene duplication, as has been reported for the *MLL-1* gene involved in acute myeloid leukemia [64], and the *BRCA1* gene and *MYB* oncogene (reviewed in [12,46]). Duplication of the *MYB* locus, which encodes an essential transcription factor, is common in human cancers. The human *MYB* locus is flanked by *Alu* repeats that are responsible for its duplication by homologous somatic recombination between adjacent *Alu* elements located on sister chromatids, as has been observed in T-cell acute lymphoblastic leukemia [65].

Recombination between *Alu* repeats is also involved in the genesis of the Philadelphia chromosome, i.e., translocation between chromosomes 9q34 and 22q11, leading to *BCR/ABL* gene fusion in chronic myeloid leukemia [66]. Similarly, translocation between chromosomes 5q23–31 and 18q12, involving the *TRE-2* oncogene, is due to recombination between *Alu* elements and plays an important role in the development of Ewing sarcoma [67]. Collectively, these data highlight the role of *Alu* repeats in mediating genome instability in human cancers.

**Table 1:** Examples of cancers and leukemias related to *Alu* and LINE-1 insertion.

Cancer or Leukemia	Altered Gene	Involved TE	Type of Event(s)	Reference(s)
Acute myeloid leukemia	*MLL1*	*Alu*	Duplication	[64]
Atypical teratoid/rhabdoid tumor	*SMARCB1*	*Alu*Y	Frameshift	[58]
Breast/ovarian cancer	*BRCA1*	*Alu*	Deletion, duplication	[12,59]
	*BRCA2*	*Alu*	Duplication, exon skipping, splicing alteration	[59,60]
Breast cancer	*NIPAL2*	LINE-1	Intronic insertion	[57]
	*MAP2K4*	*Alu*	Translocation	[57]
	*MYC*	LINE-1	Intronic insertion	[54]
Chronic myeloid leukemia	*BCR-ABL*	*Alu*	Translocation t(9;22)(q34;q11)	[66]
Colon cancer	*APC*	LINE-1	New polyA site	[20]
Ewing sarcoma	*EWSR1-ETV*	*Alu*	Translocation t(5;18)(q23–31;q12)	[67]
Hepatoma	*CAD*	*Alu*	Deletion	[61]
Hereditary diffuse gastric cancer	*CDH1*	*Alu*	Deletion	[62]
Osteosarcoma	*NCOA3*, *ZNF750*	*Alu*	Intronic insertion	[34]
Osteosarcoma	*CSMD1*, *CNTNAP2*, *ZHHX3*	*Alu* and LINE-1	Intronic insertion	[34]
Osteosarcoma	*ADAMTS9-AS2*, *CTNNA2*, *LRP5*, *NCOR1*, *OPCML*, *PLCB4*, *PRKN*, *TFNC*	LINE-1	Intronic insertion	[34]
Osteosarcoma	*KAT6A*	LINE-1	Insertion in 3′UTR-exon	[34]
Osteosarcoma	*XPC*	LINE-1	Insertion in 5′UTR-intron	[34]
T-cell acute lymphoblastic leukemia	*MYB*	*Alu*	Duplication	[65]

### 3.3. Transcriptional Activity of HERVs

HERVs have strong transcriptional promoters and enhancers, which can affect the cellular transcriptome, and encode oncoproteins, such as Rec and Np9, that can exert regulatory effects on cell proliferation and cell death balance [48,68,69]. Therefore, the transcription of HERVs, especially HERV-K, is linked to many types of cancer, such as breast cancer, melanoma, glioma, Hodgkin’s lymphoma, and prostate cancer [48,68,69,70,71,72]. For example, several HERVs loci were found transcriptionally active in Hodgkin’s lymphoma cells [70]. Unspliced HERV-H and HERV-K transcripts have been evidenced, and spliced HERV-K transcripts were detected that matched genomic sequences with the expected splicing-donor and splicing-acceptor sites [70]. Especially, HERV-K18 transcription might impact the expression of different isoforms of *CD48*, which plays multiple stimulatory and regulatory roles in the immune system [70]. Montesion et al., showed that the HERV-K promoter activity is present in the majority (73%) of breast cancer cell lines tested and that LTR sequence similarity is correlated with promoter expression patterns [71]. In addition, LTR polymorphisms at transcription factor binding sites lead to differential gene expression between individuals [71]. The activation of oncogene expression could be triggered by onco-exaptation as exemplified by the upregulation of *IRF5* driven by endogenous retroviral LTR in Hodgkin’s lymphoma cells [73]. Moreover, upregulation of the *ETV1* oncogene upon HER-V LTR producing chromosomal rearrangements may leads to prostate cancer whereas the cis promoter activation of *CSF1R* oncogene causes glioblastoma [48]. Finally, HERV-K expression has been mostly studied in breast cancer and melanoma where it could be used as a biomarker and immunological therapeutic target [69,72,74].

In addition, HERVs are also involved in the aggressiveness of tumors, and are known to be major determinants of pluripotency in human embryonic stem cells and of the reprogramming process of induced pluripotent stem cells [48]. For many tumor types, tumor aggressiveness has been associated with tumor cells obtaining stem cell characteristics. These cancer stem cells possess stem cell properties and sustain tumorigenesis. The CD133 protein is a recognized marker of cancer stem cells, and it has been demonstrated that HERV-K activation is required to expand and maintain a CD133^+^ melanoma cell subpopulation with stemness features in response to microenvironmental modifications [72].

### 3.4. TE-Mediated Epigenetic Alterations

As mentioned above, the frequent hypomethylation of chromatin in tumor cells is considered a facilitator of TE mobility [28,29,30]. Thus, it is possible to observe a correlation between the level of chromatin methylation and the rate of insertion and transcription of LINE-1 elements in cancer cells [75]. Further demonstration of the role of epigenetic regulation of TEs in cancers is supported by the study of Zhao et al. in the blind mole rat, a small rodent characterized by an unusually long lifespan (>21 years) and resistance to spontaneous and induced tumorigenesis. In these animals, cancer resistance is mediated by retrotransposable elements, and these investigators showed that the animals’ cells express very low levels of DNA methyltransferase 1 and, following cell hyperplasia, the DNA of the blind mole rat genome loses its methylation leading to activation of TEs [76]. This mechanism also works, but less strongly, in mice and humans [76]. Retrotransposition events of LINE-1 elements are common in cancers (for example 53% of patients in [32]) and LINE-1 activity, which fluctuates with tumor progression, and correlates with hypomethylation of the LINE-1 element promoter [32]. More recently, it was reported that LINE-1 hypomethylation was increasingly common with decreasing age of colorectal cancer diagnosis, suggesting a role for global DNA hypomethylation in colorectal cancer in adults younger than 55 years [77]. In addition, LINE-1 hypomethylation was associated with higher colorectal cancer-specific mortality independent of tumor molecular features and patient characteristics [77]. Despite a genome wide hypermethylation in osteosarcoma, the methylation level of *Alu* and recent LINE-1 was found to be significantly lower in tumor samples than normal osteoblast cell lines [34]. However, no clear correlation appeared between expression of TEs and methylation levels in osteosarcoma [34]. Park et al., showed that *Alu* index, i.e., quantification of *Alu* repetitions in cell-free DNA, and LINE-1 methylation level were significantly different in comparison between patients with lung or breast cancer and healthy controls, suggesting that this combination of tests could be implemented to distinguish cancer patients from healthy individuals [50]. LINE-1 methylation levels were also correlated with the human papillomavirus (HPV) status in oropharyngeal squamous cell carcinoma (OPSCC). Indeed, TEs (i.e., LINE-1, *Alu* and HERVs) were found hypermethylated in OPSCC and the 5-CpG signature distinguished HPV-positive and HPV-negative OPSCC [78]. Especially, LINE-1 methylation levels were higher in HPV-positive cases. However, the hypomethylation of promoter-associated *Alu* and LTR of HERV leads to the surexpression of ZNF541 and CCNL1 genes, respectively, which is associated with better overall survival [78]. This indicates that TEs are differentially methylated and may regulate gene expression in HPV-positive OPSCC [78].

By contrast, a tumor-suppressive role for LINE-1 has been recently described in acute myeloid leukemia. The authors showed that expression of the chromodomain protein MPP8, which is a chromatin remodeling factor, promotes LINE-1 suppression, and that enhanced LINE-1 silencing is associated with poor prognosis in human acute myeloid leukemia [79].

Escape from epigenetic control of a LINE-1 element can thus lead to cancer as shown in the following example observed in a patient with high-grade mucosal colon adenocarcinoma [80]. Failure to methylate the promoter of the specific LINE-1 locus (L1H*s*) involved, located on chromosome 17, allowed RNA transcription in normal tissue samples from the patient. This active element then caused a de novo insertion of the retrotransposon that is assumed to have occurred in the individual’s pre-cancerous colonic epithelium. This de novo L1Hs insertion in chromosome 5 disrupted an exon of the *APC* tumor suppressor gene, producing a trigger mutation that likely caused the subsequent colon cancer. The L1Hs source element on chromosome 17 is a polymorphic structural variant in human populations, and methylation studies in other individuals indicate that this specific locus can be effectively silenced. In the patient, the locus was one of about ten L1Hs that were expressed and all other loci appear to have been silenced. The reason for this specific escape from epigenetic regulation is still unknown [4].

Furthermore, DNA methylation is known to silence HERVs transcription [81], and HERV-K hypomethylation has been associated with poor prognosis in ovarian cancer [68]. Furthermore, hypomethylation in LTR promoters is able to induce carcinogenesis in B cell-derived Hodgkin’s lymphoma by deregulating the expression of oncogene protein, namely the colony-stimulating factor 1 receptor [68]. In prostate cancer cells, demethylation of the HERV-K promoter in combination with androgen stimulation induces *gag* mRNA expression [68]. This result suggested the use of combining HERV-K *gag* expression with prostate-specific antigen testing of blood samples to detect early prostate cancer, especially in high-risk men [68]. HERVs activation is also dependent of histone methylation, especially that of H3K9 by the methyltransferase SETDB1, which is overexpressed in many cancers [82]. In acute myeloid leukemia cells, the loss of *SETDB1* gene triggers desilencing of HERVs that leads to the production of double-stranded RNAs with induction of a type I interferon response and, in turn, apoptosis [83]. This suggests that HERVs silencing may be a way for cancer cells to circumvent the immune system [83].

## 4. Involvement of TEs in Non-Cancerous Pathologies and Aging

Apart from cancers, many other human diseases can have a molecular origin in chromosomal recombination, alteration of the structure and/or expression of genes in which TEs are involved (Table 2). These diseases are very diverse and include hemoglobinopathies, metabolic and neurological diseases as well as common diseases [4,12,13].

### 4.1. Implications of TEs in Hemoglobinopathies

The α-globin and β-globin gene clusters, located on chromosomes 16 and 11, respectively, are the target of numerous rearrangements leading to a group of pathologies grouped under the name of hemoglobinopathies, and including thalassemias.

Deletions involving *Alu* repeats have been observed in the β-globin gene cluster, which includes the γ-, δ-, and β-globin genes [84,85]. For example, in an individual with hereditary persistence of fetal hemoglobin (γ-globin), the β-globin cluster has a large 48.5-kb deletion downstream of the β-globin gene, and the 5′ breakpoint of the deletion is in a 3′ *Alu* element of the Aγ-globin gene, one of the two genes (Aγ and Gγ) of fetal globin. However, in this particular case, the recombination mechanism involves non-homologous recombination because the 3′ breakpoint was located in a region that contains various repeats, including part of a LINE-1 repeat, a 160-bp perfect palindrome, and a 41-bp set of direct repeats found at other locations in the human genome [86].

With respect to the α-globin gene cluster, recombination events involving *Alu*- and/or LINE-1 sequences are numerous and, overall, lead to various forms of thalassemia. The following are examples of recombination events that lead to α-thalassemia, an inherited hemoglobin disease characterized by quantitative reduction of the α-globin chain and anemia. In one family of patients with α-thalassemia, there was a 28.5-kb deletion involving *Alu* repeats that eliminated one of the duplicated α-globin genes [85]. In another patient family, an 8.2 kb deletion in the α-globin gene cluster was found. This deletion involved both α-globin genes in cis and was caused by a non-homologous recombination event between an *Alu* element and a LINE-1 element [87]. A large 33-kb deletion encompassing the α- and ζ-globin genes and pseudogenes was observed in another family and caused α^0^-thalassemia, i.e., the complete absence of the α-globin protein chain [84].

As described previously for cancer, chromatin structure and/or epigenetic regulation associated with TE insertion can lead to non-cancerous human diseases, but examples are rare to date. However, the β-globin-L1 allele results from the insertion of an entire LINE-1 element into intron 2 of the β-globin gene. The decreased transcript level of this β-globin-L1 allele is due to the hypermethylated profile of its promoter and enhancer sequences, which results in downregulation of transcription and, thus, the β^+^-thalassemia phenotype [88].

### 4.2. Implications of TEs in Metabolic Diseases and Metabolism Gene-Related Diseases

Deletions involving *Alu* repeats account for many mutations in several metabolic genes (Table 2), including the *apolipoprotein B* (*APOB*) gene and the *LDL receptor* (*LDLR*) gene. For example, in a patient with homozygous hypobetalipoproteinemia, the deletion of exon 21 of the *APOB* gene is the consequence of a recombination event between the *Alu* sequences of introns 20 and 21, resulting in a nonreciprocal exchange between two chromosomes [89]. The *LDLR* gene is also a locus of large deletions (or duplications) by homologous recombination between *Alu* repeats, which are numerous in almost all introns of the gene and in the 3′ untranslated region (3’UTR) of the mRNA [90]. For example, analysis of autosomal dominant hypercalcemia in two patient families showed the presence of a large deletion in the *LDLR* gene due to non-allelic intra-chromosomal homologous recombination between two homologous *Alu* sequences located away from the splice sites in the intronic sequences [91]. Furthermore, the LINE-1 element can also cause deletions as is the case for the *pyruvate dehydrogenase E1* (*PDHX*) gene located on the X chromosome. In this case, a large 46 kb deletion linked to the insertion of a full-length LINE-1 element led to a pathological deficiency of the pyruvate dehydrogenase complex [92]. However, insertion of LINE-1 into the *PDHX* gene can also lead to an aberrantly spliced isoform resulting from the use of two cryptic splice sites [92].

Insertion of *Alu* or LINE-1 elements can also lead to the creation of a new exon, or exonization (Table 2). For example, a G-to-C mutation in an *Alu* element present in intron 3 of the *ornithine delta-aminotransferase* (*OAT*) gene resulted in the creation of a new splice donor site and potentially a new exon [93]. This insertion of an *Alu* element also provides a premature transcription termination site and also results in an *OAT* deficiency. In both cases, this *Alu* element insertion is involved in mitochondrial *OAT* deficiency that causes hyperornithinemia leading to chorioretinal gyrate atrophy, a disease characterized by myopia and night blindness progressing to progressive vision loss. Similarly, exon creation due to insertion of an *Alu* element has been observed in the β-*glucuronidase* gene (*GUSB*) that causes Sly syndrome or mucopolysaccharidosis type VII [94]. The LINE-1 retroelement is also a potential source of exon creation, implicated in at least one case of chronic granulomatous disease, a condition that promotes bacterial and fungal infections [95]. In this case, the X-linked *CYBB* gene encoding the cytochrome B245 beta chain has an insertion of LINE-1 in intron 5, causing internal rearrangements and new splice sites that result in a highly heterogeneous splicing pattern with the introduction of two LINE-1 fragments as new exons in mRNAs and exon skipping [95].

### 4.3. Implications of TEs in Neurological and Psychiatric Diseases

TE insertion can cause neurological diseases or syndromes [24,96] (Table 2). The insertion of a LINE-1 element in the *ribosomal S6 kinase 2* (*RSK2*) gene was reported in a patient with Coffin-Lowry syndrome, a disease characterized by psychomotor and growth retardation, facial dysmorphism, and skeletal abnormalities. This LINE-1 insertion at position 8 of intron 3 leads to a skipping of exon 4, followed by a reading frame shift and the appearance of a premature stop codon [97]. Ataxia telangiectasia is a rare inherited disorder that affects the nervous system, immune system, and other body systems and which is characterized by progressive difficulty with coordinating movements. An increase in human-specific LINE-1 DNA copy number has been observed in postmortem brain tissue derived from ataxia telangiectasia patients compared with healthy controls [37].

Fabry disease, characterized by central nervous system neurodegeneration, is an X-linked recessive disorder of glycosphingolipid catabolism that results from deficient activity of the lysosomal hydrolase α-galactosidase A encoded by a gene rich in *Alu* repeats. Indeed, this gene has twelve *Alu* elements in its 12-kb sequence [98]. Several mutations and gene rearrangements have been described as causing the disease, of which a single deletion was originally identified as a recombination between *Alu* repeats and ten others as rearrangements mediated by *Alu* repeats [98]. Subsequently, a large 3.1 kb deletion due to *Alu-Alu* recombination and including the entire exon 2 was identified among 50 novel Fabry disease-causing mutations [99].

Sandhoff disease, a recessive lysosomal storage disease, is another example of a TE-related neurodegenerative disease. Deficiency in β-hexosaminidase (HEXB) activity is due to deletion alleles of the *HEXB* gene in 27% of the dataset examined [100]. The *HEXB* gene contains two *Alu* repeats that are involved in the deletion event, which removes approximately 16 kb, including the *HEXB* gene promoter, exons 1 through 5, and part of intron 5. The deletion also created a reconstituted *Alu* sequence, with the left half coming from the 5′-*Alu* sequence and the right half from the 3′-*Alu* sequence [100].

Neurofibromatosis type I is a common genetic disease linked to the *NF-1* tumor suppressor gene and characterized by changes in skin coloration and the growth of benign tumors along the nerves, i.e., neurofibromas. Insertion of an *Alu* sequence into the intron of the *NF-1* gene results in deletion and frameshift in the downstream exon upon splicing, which may be a cause of the disease [101].

High expression of TEs has been observed in several human neurodegenerative diseases, including sporadic Creutzfeldt-Jakob disease, age-related macular degeneration, and amyotrophic lateral sclerosis (ALS) [96]. For example, the RNA-binding protein TDP-43, which is implicated in ALS and frontotemporal lobar degeneration (FTLD), interacts with many TE transcripts, and this association of TDP-43 with its target TE RNAs is reduced in FTLD patients [102]. As mentioned previously, insertion of a SVA element into an intron of the *TAF1* gene causes XPD [19] (Figure 3). The insertion prevents normal mRNA splicing and results in retention of intron 32 [18]. Furthermore, this SVA insertion specifically reduces acetylated histone H3 binding on exon 32, which is closest to the insertion. Conversely, excision of the SVA by CRISPR/Cas9 technology restores acetylated histone H3 binding [103].

By using two independent murine models for endogenous retrovirus (ERV) activation, namely intracerebroventricular injection of streptozotocin and the muMT strain lacking B cells and antibody production, Sankowski et al. found that induced hippocampal ERV activation was accompanied by a significant hippocampus-related memory impairment in both models [104]. These results suggest a role for ERVs as endogenous drivers of cognitive deficit in humans. In addition, this study also showed that cognitive deficits are attenuated in the absence of mitochondrial antiviral signaling proteins, which are retroviral RNA-sensing proteins, suggesting that these proteins may be therapeutic targets to treat dementia and neuropsychiatric disorders [104].

A link between TEs and psychiatric disorders has also been demonstrated [105,106,107]. In particular, whole-genome sequencing revealed brain tissue-specific insertions of LINE-1 in patients, preferentially located in synapse-related and schizophrenia-related genes, suggesting a causal role of LINE-1 in disease development [107,108]. Moreover, expression of HERVs in blood and cerebral spinal fluid was observed in individuals with schizophrenia [107,109]. The involvement of TEs was also reported for affective disorders [110], stress-related psychiatric disorders [107,111], and post-traumatic stress disorder [107]. One study also found hypomethylation of LINE-1 in bipolar individuals [108]. Finally, the involvement of TEs has been reported in several cases of autism spectrum disorder (ASD) [107]. The increased expression of LINE-1 has been observed in post-mortem cerebellum of ASD individuals compared to matched controls but not in the three other cortical brain regions studied [112]. The creation of a permissive state for LINE-1 activity in the cerebellum in ASD could be due to decreased binding of methyl-CpG-binding protein 2 to the 5′UTR of LINE-1, leading to ORF 1 downregulation accompanied by reduced H3K9me3 levels in the cerebellum in the ASD group but not in healthy controls [112]. LINE-1 was also shown to be hypomethylated in lymphoblastoid cells derived from a subset of ASD patients [113]. In addition, *Alu* methylation status was shown to vary by ASD subtype, suggesting a role for *Alu* elements [114].

Finally, major depressive disorder, commonly known as depression, is a debilitating but common illness with both genetic and environmental risk factors. It has been hypothesized that exposure to either exogenous viruses or traumatic experiences can activate HERVs in the brain to cause depressive symptoms [115]. In addition, a second hypothesis is that individual differences in vulnerability or resilience result from the balance of activated HERVs with pathogenic versus protective functions in the brain [115]. However, these hypotheses have to be demonstrated experimentally.

### 4.4. Implications of TEs in Various Other Diseases

Alveolar capillary dysplasia with misalignment of pulmonary veins is a rare and lethal neonatal developmental lung disorder caused by point mutations or copy-number variant deletions of the *FOXF1* gene, or its distant tissue-specific enhancer, often due to the presence of TE insertion. Indeed, breakpoints sequencing of causative deletion showed that half are *Alu*-mediated deletion and 30% displayed a LINE-1 sequence at least at one of the two breakpoints [116]. The *FOXF1* locus at 16q24.1 is a large genomic instability hotspot including two evolutionary young LINE-1, namely L1PA2 and L1PA3, flanking five *Alu* sequences (i.e., *Alu*Y, two *Alu*Sx, *Alu*Sx1, and *Alu*Jr) related to the disease [116].

Alternative splicing is often affected by insertion of TEs and, for example, insertion of the *Alu* element into an intron of the *human factor VIII* (*F8*) gene leads to exon skipping and, consequently, to the development of hemophilia A [117]. Another example is the insertion of an *Alu* element in exon 9, or just at the beginning of exon 9, of the *fibroblast growth factor receptor 2* (*FGFR2*) gene, which may be a cause of Apert syndrome [118]. The latter mutation affects the use of the 3′ splice site, leading to the genesis of different nonfunctional splice forms of *FGFR2* gene transcripts. By contrast, exon creation following insertion of an *Alu* element in the *type IV collagen alpha-3 chain* gene (*COL4A3*) may be the cause of Alport syndrome, a nephropathy (glomerulonephritis) due to a defect in collagen IV [119].

The *dystrophin* gene (*DMD*), located on chromosome X, is a giant gene of about 2.5 Mb in size with more than 60 exons. Numerous TE insertions into the *DMD* gene have resulted in deletions or exon skipping. For example, the insertion of LINE-1 induces the skipping of exon 44, which causes Duchenne muscular dystrophy [120]. Insertion of an *Alu*-like sequence downstream of intron 11 of the *DMD* gene has been observed in individuals with X-linked dilated cardiomyopathy [121]. This insertion induces a rearrangement that activates a cryptic splice site in intron 11 and produces an alternative transcript containing the *Alu*-like sequence and part of the adjacent intron 11, spliced between exons 11 and 12 but not translated because of the presence of many stop codons [121]. Other examples of exon skipping due to *Alu* or LINE-1 insertion include the *OPA1* gene involved in autosomal dominant opticatrophy [122], the *FKTN* gene responsible of Fukuyama-type congenital muscular dystrophy [123], and the *IDS* gene involved in Hunter syndrome [124] (Table 2). Alternative splicing alteration and an open reading frame shift are also involved in X-linked retinitis pigmentosa owing to LINE-1 insertion in the RP2 gene [125].

In addition, several diseases have been linked to the introduction of a novel polyadenylation site by insertion of LINE-1 or *Alu* elements into human genes, such as hemophilia A and B [126], autoimmune lympho-proliferative syndrome [127], X-linked dilated cardiomyopathy [121], hypocalciuric hypercalcemia [128], and severe neonatal hyperparathyroidism [128] (Table 2).

The increased expression of TEs may also promote the recruitment of inflammatory processes and the disruption of immunological balance, which can lead to chronic inflammation in idiopathic pulmonary fibrosis (IPF) [129]. Indeed, these authors observed TE dysregulation in the alveolar type II lung cells and alveolar macrophages of IPF patients and the increased TE, especially LINE-1, expression is positively correlated with both the activation of cellular TE inhibitors and the innate immune response and negatively correlated with autophagy in IPF [129]. The involvement of HERVs in innate immunity has been also demonstrated and HERVs contribute to pro-inflammatory diseases such as Type-I diabetes, autoimmune disorders and multiple sclerosis, as well as neuropathogenesis of severe acute respiratory syndrome coronavirus-2 (SARS-CoV-2) [130]. Finally, the mutual interaction between microbiote and HERVs expression was highlighted in humans by a strong correlation between the expression of HERV-H, -K and -W, and the concentration of *Bifidobacterium* spp. in the gut [130,131]. Moreover, the expression of ERVs was found to be favored by the skin microbiota as shown by using mouse keratinocyte model challenged by various skin commensals [132].

**Table 2:** Examples of non-cancerous diseases related to TE insertion.

Disease or Syndrome	Altered Gene	Involved TE	Type of Event(s)	Reference(s)
Alport syndrome	*COL4A3*	*Alu*	Exonization	[119]
Alveolar capillary dysplasia with misalignment of pulmonary veins	*FOXF1*	*Alu*, LINE-1	Deletion	[116]
Apert syndrome	*FGFR2*	*Alu*	Exon skipping, aberrant splicing	[118]
Autoimmune lympho-proliferative syndrome	*FAS*	*Alu*	Exon skipping, new poly(A) site	[127]
Autosomal dominant opticatrophy	*OPA1*	*Alu*	Exon skipping	[122]
Chronic granulomatous disease	*CYBB*	LINE-1	Exonization, intron retention	[95]
Coffin-Lowry syndrome	*RPS6KA3*	LINE-1	Exon skipping, frameshift	[97]
Duchenne Muscular Dystrophy	*DMD*	LINE-1	Exon skipping	[120]
Fabry disease	*a-GALA*	*Alu*	Deletion	[98,99]
Familial hypercholesterolemia	*LDLR*	*Alu*	Deletion	[91]
Fanconi anemia	*UBE2T*	*Alu*	Deletion, duplication	[14]
Fukuyama-type congenital muscular dystrophy	*FKTN*	LINE-1	Exon skipping	[123]
Hemophilia A	*F8*	*Alu*	Exon skipping	[117]
Hemophilia B	*F9*	*Alu*, LINE-1	New poly(A) site	[126]
Hunter syndrome	*IDS*	*Alu*	Exon skipping	[124]
Hypo beta lipoproteinemia	*APOB*	*Alu*	Deletion	[89]
Hypocalciuric hypercalcemia and neonatal severe hyper-parathyroidism	*CASR*	*Alu*	New poly(A) site	[128]
Multiple sclerosis	*CD58*	*Alu*	Exon skipping, frameshift	[133]
Neurofibromatosis type 1	*NF1*	*Alu*	Aberrant splicing, frameshift	[101]
Opitz syndrome	*MID1*	HERV-E	New regulatory region	[21]
Ornithine delta-aminotransferase deficiency	*OAT*	*Alu*	Exonization	[93]
Pyruvate dehydrogenase complex deficiency	*PDHX*	LINE-1	Aberrant splicing, deletion	[92]
Sandhoff disease	*HEXB*	*Alu*	Deletion	[100]
Sly syndrome	*GUSB*	*Alu*	Exonization	[94]
Thalassemia	*HBB*	*Alu*	Deletion, promoter hypermethylation	[84,85,86,87,88]
Von Hippel Lindau disease	*VHL*	*Alu*	Deletion	[63]
X-linked dilated cardiomyopathy	*DMD*	*Alu*, LINE-1	New poly(A) site, aberrant splicing	[121]
X-linked dystonia with parkinsonism	*TAF1*	SVA	Intron retention	[18,19]
X-linked retinitis pigmentosa	*RP2*	LINE-1	Aberrant splicing, frameshift	[125]

### 4.5. TEs and Common Diseases Relationship

The increase in genomic studies and, in particular, Genome-Wide Association Studies (GWAS) strengthens the case for a genetic basis for common diseases, i.e., diseases that have a high incidence in the population (>1:2000) and are not usually classified as hereditary diseases. However, these diseases often have a genetic basis and a family aggregation can be observed. They are called multifactorial diseases since, in addition to genetic predisposition factors, a complex set of environmental factors (food, viral and bacterial infections, other diseases, exposure to pollutants, smoking, alcohol and drugs) affect development of the pathology. These common diseases are very varied and include cardiovascular diseases (hypertension, myocardial infarction, among others), endocrine diseases (type 2 diabetes, obesity and hypercholesterolemia), osteoarticular pathologies (osteoarthritis, osteoporosis) neurological diseases (senile dementia), dysimmune and autoimmune diseases (e.g., multiple sclerosis), psychiatric diseases (schizophrenia, bipolar disorder, autism) and allergy/asthma problems. Hundreds of different insertions of *Alu* elements have been identified in the best-defined predisposition loci or genes for these diseases [134]. Furthermore, in many cases, the mobile element insertion is in linkage disequilibrium with disease haplotype markers, i.e., the *Alu* element appears linked to single nucleotide polymorphisms (SNPs) associated with the disease. This suggests that the TE insertion is likely to be associated with, or even cause, the genetic risk factor for developing the disease in question.

An example is the insertion of an *Alu* element at the *CD58* locus on a haplotype that affects susceptibility to multiple sclerosis (MS). Of the many risk alleles identified by GWAS for this disease, the *CD58* genotype is one of the strongest genetic indicators of MS risk. The risk allele reduces the expression of the *CD58* gene and this reduction in *CD58* expression is associated with the risk of developing MS and relapsing. In-depth sequence study of this locus showed a very strong genetic link between a 302 bp *Alu* insertion and a particular SNP (namely rs2300747). Only this allele of the *CD58* locus with both the *Alu* insertion and the rs2300747 SNP is associated with an increased risk of developing the disease. This *Alu* insertion is relatively frequent since it is present in 66% of individuals. Furthermore, this *Alu* sequence insertion alters *CD58* mRNA splicing, promoting exon 3 skipping and a reading frame shift leading to a non-functional mRNA [133]. These observations thus show that routine insertion of an *Alu* sequence compromises *CD58* gene expression, creating an allele that increases susceptibility to MS. More generally, it can thus be concluded that inherited TE insertion alleles, which are common to all our genomes, can modify the risk of developing many types of common disease.

### 4.6. TEs and Aging

Finally, TE transposition can lead to mutations and damage to DNA. Double-strand breaks, which can be caused by transposition [33], are one of the processes directly related to aging. Thus, TEs can be considered an internal source of aging and the frequency of transposition can, in turn, affect the rate of aging [23]. The PIWI-piRNA pathway appears as the main link between TEs and aging. Although the primary mechanism for this connection appears to be repression of transposition-generated double-strand breaks, PIWI-piRNA-directed DNA methylation and changes in PIWI-piRNA-associated histone modifications are two other mechanisms linking the PIWI-piRNA pathway and aging [23]. Nevertheless, further experiments are needed to determine the extent of the role of TEs relative to other sources of DNA damage or different causes of aging. The putative role of TEs in aging is further supported by a recent study in mice, which shows that TE expression increases with age in the brain of 20-month-old mice [135]. Of the differentially expressed TEs in the mouse brain at twenty months, 92.7% increase with age. The LTR family is particularly affected and ERV is the predominant family of LTR retrotransposons that increases with age [135]. In addition, the longevity regulatory protein, SIRT6, appeared as a potent silencer of LINE-1 in mice, and its depletion during aging, or following DNA damage, allows the reactivation of LINE-1 [136]. Specifically, SIRT6 binds to the 5’UTR of LINE-1 and causes mono-ADP ribosylation of the corepressive nuclear protein, KAP1, which facilitates the interaction of KAP1 with the heterochromatin factor, HP1a, and thus contributes to the enclosure of LINE-1 elements into the transcriptionally repressive heterochromatin [136]. The increase of TEs expression or TE activation, especially that of LINE-1 and HERVs, may be broadly impacted by Alzheimer disease and/or Tau pathologies in human brains [137,138,139].

## 5. Role of Stress and Environmental Pollution in TE Mobility and Disease Onset

In plants and animals, different forms of stress are known to act as triggers or facilitators of TE mobility [140,141]. Several studies in plants have reported overexpression of TEs following abiotic or biotic stress conditions such as temperature, nitrate deprivation, and wounding [142,143,144,145]. Activation of TEs in response to different stresses has also been demonstrated in insects [146], and especially in the context of insecticide resistance [147,148,149]. This suggests that human exposure to pesticides and other environmental pollutants could act on the mobility of TEs in humans and be the cause of certain pathologies such as cancers.

To date, there is no direct demonstration of a causal relationship between exposure to a pollutant, TE mobility and development of a pathology. However, the arguments in favor of this hypothesis are of two types: (i) it has been shown experimentally or in population studies that pesticides and other environmental contaminants can modify the different levels of epigenetic regulation and thus create a context favorable to the mobility of TEs; (ii) mobility tests conducted on human or rodent cell models have shown that exposure to different contaminants or stresses can promote the mobility of TEs, in particular of LINE-1 elements [150].

### 5.1. Epigenetic Alterations Caused by Environmental Pollutants

Pesticides of various classes can cause epigenetic modifications such as DNA demethylation and histone modification. Several animal studies have shown hypomethylation of certain promoters after exposure to high doses of pesticides (e.g., organochlorines, biphenyl-polychlorines, methyl-mercury). In addition, dichlorodiphenyltrichloroethane (DDT) is known to modify the DNA methylation profile, in particular in the brain of young rats where DDT-induced hypomethylation suggests a role for this pesticide in neurodegenerative diseases [151]. Metoxychlor, an organochlorine pesticide acting as an endocrine disruptor also alters methylation patterns leading to reproductive defects in female offspring [152]. The methylation of all the genes tested is affected, and these changes are transmitted to the offspring because only the gametes are affected, but they gradually disappear between the first and third generation [152]. The endocrine disruptor di(2-ethylhexyl)phthalate (DEHP) interferes with sex hormones signaling pathways and induces testicular dysgenesis syndrome depending on the mouse strain [153]. This transgenerational endrocrine disruption effect could be explained by the existence of SNP-dependent mechanisms and a DHEP-induced increase of the Svs3ab promoter methylation persisting across generations [153]. Previously, DEHP was reported to induce an increase in mir-615 microRNA expression and a genome-wide decrease in microRNA promoter methylation as well as methylation-associated silencing of almost the entire cluster of the seminal vesicle secretory proteins and antigen genes, which are known to play a fundamental role in sperm physiology [154].

In humans, global DNA hypomethylation, as measured by the % of 5-methyl-Cytosine at the *Alu* and LINE-1 sequences in adipocytes where xenobiotics are concentrated, has been linked to low-dose exposure to organochlorine pesticides in healthy individuals [155]. The same correlation between levels of persistent organic pollutants (DDT, DDE, β-BHC, oxychlordane, mirex) or polychlorinated biphenyls in blood and overall DNA hypomethylation was observed in another population [156]. Results from a cohort study showed that prenatal exposure to persistent organic pollutants (POPs), including organochlorine pesticides, polybrominated diphenyl ethers, and polychlorinated biphenyls, alter the DNA methylation level of LINE-1 and imprinted genes in placenta [157]. Especially, elevated concentrations of β-hexachlorhexane in maternal serum collected during delivery were significantly associated with a decrease in methylation of LINE-1 in the placenta [157]. A cross-sectional study was conducted using the data of 444 individuals (253 men and 191 women) exposed to sixteen different POPs, including six organochlorine pesticides and ten polychlorinated biphenyls. The correlation between POPs level in serum and DNA methylation of *Alu* and LINE-1 in peripheral leukocytes was assessed and the results showed that several POPs were associated with global DNA hypomethylation in the *Alu* assay for men and global DNA hypermethylation in the LINE-1 assay for women [158].

Exposure to pesticides may increase the risk of cancers, perhaps mediated in part through global alterations of DNA methylation. In order to evaluate alterations of LINE-1 methylation by pesticides in a variety of classes, data from 596 cancer-free male participants enrolled in the Agricultural Health Study (AHS) were used to examine associations between use of 57 pesticides and LINE-1 methylation in peripheral blood leucocytes [159]. The results of this study showed that prior application of 10 pesticides was positively associated and prior application of eight pesticides was negatively associated with LINE-1 methylation. In dose-response analyses with specific pesticides, increase in LINE-1 methylation was observed with increases in five pesticides, namely imazethapyr, fenthion, S-ethyl-*N*,*N*-dipropylthiocarbamate, butylate, and heptachlor, whereas a decrease in LINE-1 methylation was noticed with increases in three pesticides, namely carbaryl, chlordane, and paraquat [159].

Comparing LINE-1 methylation in 50 rural sprayers and 50 urban workers exposed to unspecified pesticides, the authors observed a more pronounced hypomethylation of LINE-1 for most genotypes, and hypermethylation of some heterozygous genotypes in the urban group compared with the rural group [160]. This suggest that urbanization could play an additional risk for epigenetic changes associated with pesticide exposure [160]. Arsenic is also a common pollutant in the waters of some countries and its impact on methylation, especially at the level of LINE-1 elements, has been evaluated in many studies, sometimes contradictory [161]. Therefore, modification of the DNA methylation rate and in particular the hypomethylation of the *Alu* and LINE-1 sequences by pesticides and other pollutants represents a very probable mechanism of reactivation of TEs and consequently of induction of pathologies such as cancers [12,43].

Furthermore, histone modification is also a possible mechanism of human pesticide toxicity. In particular, an increase in histone acetylation (H3 and H4) has been shown following treatment of neuronal cells with the herbicide Paraquat and the insecticide Dieldrin [162,163]. The organochlorine pesticide chlordecone, which displays well-recognized estrogenic properties, was also reported to modify the methylation level of histone H3 on lysine 4 and 9 (H3K4m3 and H3K9me3, respectively) in human cord blood and human cord KE-37 cell line [164].

### 5.2. Methylation Status of LINE-1 as a Marker of Environmental Pollution

The methylation status of LINE-1 compared to that of specific genes is often used as a marker of epigenetic alteration following pollutant exposure. For example, strong ligands of the aryl hydrocarbon receptor (AhR) such as such as 6-formylindolo [3,2-b]carbazole, 3-methylcholantrene and benzo[a]pyrene are known to induce hypomethylation of LINE-1 and then increase LINE-1 mRNA expression through the mitogen-activated protein kinase (MAPK) in breast cancer cell line and through the transforming growth factor-β1 (TGF-β1)/Smad pathway in HepG2 hepatocarcinoma cell line [165]. Exposure to weak AhR ligands such as hexachlorobenzene and chlorpyrifos also reduce LINE-1 methylation levels and induce LINE-1 reactivation, i.e., increased mRNA expression, in both MDA-MB-231 breast cancer cells and non-tumoral breast epithelial cells (i.e., NMuMG cell line) [165]. However, the basal expression of LINE-1 was significantly higher in the MDA-MB-231 cell lines compared to NMuMG non-tumoral cells [165]. In addition, the decrease of H3K9me3 level in chlordecone-exposed samples was associated with a decreased methylation in LINE-1 promoters, increased presence of H3K4me3, and then increased expression of LINE-1 ORF-1 and -2 [164].

### 5.3. Mobility of LINE-1 Elements in Response to Environmental Pollution

The use of mobility assays for LINE-1 elements using cells transfected with modified LINE-1 elements containing a reporter gene and the estimation of the copy number are valuable approaches to assess the impact of a stress or an environmental pollutant on the activity of LINE-1. These approaches, which are still not widely used, are being developed and allow us to conclude that environmental pollutants have a strong impact on the activity of LINE-1 elements and, by extension, on all TEs. Thus, many pollutants have been tested for their ability to activate the mobility of LINE-1 elements in different human or animal cellular models [150]. It was found that metals such as aluminum, arsenic, cadmium, mercury and nickel cause an increase in LINE-1 retrotransposition events, whereas cobalt, lead, magnesium and zinc have no effect. Copper even seems to have an inhibitory effect [166]. However, it is important to note that these metals were tested alone and that it may be interesting to analyze the effects of mixtures since this is what humans are most often exposed to. Carcinogens such as benzo[a]pyrene, phorbol esters, or heterocyclic amines also seem to be able to induce the mobility of LINE-1 elements although the results, obtained on mouse models, are not necessarily generalizable to humans [150].

## 6. Conclusions

Initially considered as junk DNA, TEs are now recognized as major players in genome evolution, and their role in genome plasticity has been clearly demonstrated. The high abundance of TEs in the human genome, in particular the *Alu* and LINE-1 repeats, makes them responsible for the molecular origin of several diseases. This involves several molecular mechanisms, in addition to the insertion of TEs per se, including DNA recombination and chromosomal rearrangements (especially deletion); modification of gene expression by introduction or modification of poly-A sites, splice sites, exons or introns and regulatory elements, as well as alteration of epigenetic regulations. Therefore, improving our knowledge of TEs through deep genomic approaches may lead to new potential diagnostic markers of diseases and prenatal markers of genetic diseases. Thus, the development of combined molecular (i.e., next generation sequencing and whole genome sequencing) and bioinformatics tools is of great interest despite mostly used in the cancer field [32,34,50,55,56,113,130,167,168,169]. Finally, the exposure of individuals to numerous stresses and in particular to environmental pollutants and contaminants seems to have a non-negligible impact on the epigenetic derepression and mobility of TEs, which can lead in this way to the development of certain pathologies such as cancers, neurological or autoimmune diseases.

## Data Availability

Not applicable.
